# Novel mutations underlying argininosuccinic aciduria in Saudi Arabia

**DOI:** 10.1186/1756-0500-3-79

**Published:** 2010-03-18

**Authors:** Faiqa Imtiaz, Moeen Al-Sayed, Danyah Trabzuni, Bashair R Al-Mubarak, Osama Alsmadi, Mohamed S Rashed, Brian F Meyer

**Affiliations:** 1 Saudi Diagnostics Laboratory, King Faisal Specialist Hospital & Research Centre, PO Box 3354, Riyadh 11211, Saudi Arabia; 2 Department Of Medical Genetics, King Faisal Specialist Hospital & Research Centre, PO Box 3354, Riyadh 11211, Saudi Arabia; 3 National Laboratory for Newborn Screening, King Faisal Specialist Hospital & Research Centre, PO Box 3354, Riyadh 11211, Saudi Arabia

## Abstract

**Background:**

Argininosuccinic aciduria (ASAuria) is an autosomal recessive disorder of the urea cycle relatively common in Saudi Arabia as a consequence of extensive consanguinity. It is the most common urea cycle disorder identified in the Saudi population, which therefore prioritizes the need to delineate the underlying molecular defects leading to disease.

**Findings:**

We utilized Whole Genome Amplification (WGA), PCR and direct sequencing to identify mutations underlying ASAuria cases diagnosed by our institution. A missense mutation that accounts for 50% of Saudi ASAuria patients was recently reported by our laboratory. In this study we report a further six novel mutations (and one previously reported) found in Saudi patients with ASAuria. The novel four missense, one nonsense and one splice-site mutation were confirmed by their absence in >300 chromosomes from the normal population. Pathogenicity of the novel splice-site mutation was also confirmed using reverse transcriptase-PCR analysis. Cross species amino acid conservation at the substituted residues described were observed in some but not all instances.

**Conclusions:**

Together, the eight mutations described by our laboratory, encompass >90% of ASAuria patients in Saudi Arabia and add to about 45 other ASAuria mutations reported worldwide.

## Introduction

Argininosuccinic aciduria (ASAuria; OMIM 207900) or argininosuccinate lyase (ASL; EC 4.3.2.1) deficiency is an autosomal recessive inborn error of the urea cycle. ASL catalyses the reversible cleavage of argininosuccinic acid (ASA) into arginine and fumarate. ASL is a homotetramer of 50-kDa subunits and is found in the cytosol of hepatoctyes, renal cells and fibroblasts. Neonatal presentation of ASAuria is most common and is characterized by lethargy, vomiting, hypothermia, decreased consciousness and coma usually occurring between 24 and 72 hrs. Biochemical analysis reveals hyperammonemia, elevated plasma citrulline levels and increased ASA in dried blood spots (DBS), plasma and urine[1].

The *ASL *gene is located on chromosome 7q11.2, and is approximately 35 kb in length and spans 17 exons (16 coding). Mutations in *ASL *have been shown to cause ASAuria but due to substantial clinical and genetic heterogeneity reported in this disease, a clear genotype/phenotype correlation has not been clearly defined [2-10]. Recently, an *ASL *pseudogene was reported to be located in the centromeric region of chromosome 7 [11].

ASAuria is the most common inborn error of the urea cycle in Saudi Arabia, a consequence of extensive consanguinity. It is diagnosed clinically and confirmed biochemically by the elevated levels of ASA and citrulline in dried blood spots (DBS), using tandem mass spectrometry (MS/MS) [12]. Recently, our laboratory reported that a novel missense mutation, Q354X (c.1060C>T), was found to be exclusively homoallelic in 50% of ASA patients that were screened [13]. In this paper, we report the identification of a further six novel mutations, that in combination with a previously reported mutation and Q354X provide above 90% coverage of the mutations underlying ASA in Saudi Arabia.

## Materials and methods

### Patients

All but one of the patients being reported here were diagnosed by our newborn screening laboratory based on elevated ASA and citrulline by MS/MS analysis of DBS. The samples were referred to the laboratory from sick neonates where a diagnosis of urea cycle disorder was suspected. No additional clinical details are available on these patients. Waiver of informed consent was provided by the Institutional Review Board (IRB) of the King Faisal Specialist Hospital and Research Centre (KFSH&RC), on the basis of anonymization of all study samples. The whole blood of one clinically diagnosed affected individual (and her parents) was also screened as part of this study via the molecular genetic testing services provided by the Saudi Diagnostics Laboratory for KFSH&RC. As these samples were submitted for routine diagnostic purposes in the same hospital, the IRB in place, covering these samples remains applicable.

### Sample collection and DNA extraction

Genomic DNA was extracted from whole blood by standard salt-precipitation methods. DNA was prepared from DBS previously used for MS/MS analysis. Briefly, a sample 2 mm in diameter was punched out from each spot and placed in a 96-well plate. The 2 mm discs were re-hydrated with 10 μl of phosphate-buffered saline (PBS) at room temperature prior to DNA extraction and amplification. Extraction and amplification were carried out as described for the REPLI-g TM kit (Molecular Staging Inc., New Haven, CT, USA) with minor modifications. Extractions were assembled on ice by adding 10 μl of cell lysis solution (400 mM KOH, 10 mM EDTA at pH 8.0, and 50 mM dithiothreitol), mixed briefly with the PBS-soaked discs and incubated for 10 min. Then 10 μl of neutralization solution (800 mM Tris-hydrochloride) was added and mixed gently by pipetting. This lysate mixture (2 μl) was then used as template per 10 μl of whole genome amplification (WGA) reaction.

### WGA

WGA [14] was performed in a total volume of 10 μl. Amplification reactions were assembled in 96-well plates by mixing 2.5 μl 4× buffer, 5.4 μl ddH_2_O, 0.1 μl ø29 DNA polymerase, and 2 μl of the lysate mixture. Reactions were incubated at 30°C for 16 h and terminated by heating to 65°C for 3 min. The amplification reactions were diluted 50 fold in ddH_2_O (DNA concentration ~10 ng/μl) and dilutions used in PCR amplification and direct sequencing.

### Mutation Detection

For sequence analysis of blood spot samples, WGA generated DNA was amplified by PCR using intronic primers designed to amplify the 16 coding exons of the *ASL *gene (primer sequences and conditions are available on request). PCR was performed in a final volume of 20 μl containing approximately 10 ng of genomic DNA, 50 mM KCl, 10 mM Tris-HCl (pH 8.3), 1.5 mM MgCl_2_, 100 μM deoxyribonucleotide triphosphates (dNTPs), 1 unit of Qiagen (Valencia CA, USA) HotStar Taq polymerase, and 50 ng of each primer. Thermocycling (Applied Biosystems Inc, Foster City, CA, USA) consisted of an initial denaturation at 95°C for 15 min followed by 35 cycles of PCR. Each cycle of PCR consisted of denaturation at 94°C for 60 s, annealing at 62-68°C for 60 s and extension at 72°C for 60 s. A final extension step of 10 min at 72°C was added. Sequencing reactions were performed using an ABI Prism Big Dye Terminator v3.1 Cycle Sequencing Kit following the manufacturer's instructions and were processed on a MegaBACE 1000 DNA Analysis System (Molecular Dynamics; Sunnyvale, CA, USA). Sequence analysis was performed using the SeqMan 6.1 module of the Lasergene (DNA Star Inc. WI, USA) software package, then compared to the reference GenBank sequence (accession no. AF376770). Numbering commenced with the A of the ATG initiation codon as +1.

### RT-PCR

To determine the effect of the novel splice-donor site mutation detected in intron 13 with respect to mRNA splicing, a fragment comprising exon 12, 13 and 14 of *ASL *was amplified using RT-PCR with mRNA isolated from peripheral blood lymphocytes. One microgram of total RNA from the patient and a normal control was reverse transcribed using an aliquot of a reverse transcription reaction containing 1 × PCR buffer, 2.5 mM MgCl_2_, 0.2 mM of each dNTP, and 0.5 U AMV reverse transcriptase (Roche Diagnostics GmbH, Mannheim, Germany). 10 μl of cDNA was amplified by PCR using the 5' primer (GGC TCC TGA TGA CCC TCA A) and the 3' primer (ATA GGC AAG GTC AGT GGC CAG). Thermocycling consisted of an initial denaturation at 94°C for 10 min followed by 35 cycles of PCR at 94°C for 60 s, 64°C for 60 s and extension at 72°C for 60 s.

### URLs

We used the Ensembl website http://www.ensembl.org to obtain exon and intron boundaries and the reference GenBank cDNA sequence, and Primer3 http://frodo.wi.mit.edu/primer3/ to design appropriate primers.

## Results

Twenty-nine patients were studied, 15 (54%) were homoallelic for the previously reported Q354X mutation. Direct sequencing of *ASL *gene in the remaining 13 patients revealed the presence of seven mutations (4 missense, 2 nonsense and 1 splice-site). The next most common mutation was a novel transition c.556C>T in exon 7 and resulted in the substitution of arginine for tryptophan (R186W). Eight patients (29%) were homozygous for R186W and one patient was found to be a compound heterozygote having R186W in combination with a transversion, c.343G>T in exon 4 which resulted in the novel substitution of aspartic acid for tyrosine at position 115 in the amino acid sequence (D115Y). A homozygous transition c.469G>A in exon 6 was found in one patient and resulted in the previously unreported substitution of glycine for arginine at position 157 (G157R). The fourth novel missense mutation, P166S, observed in one patient was caused by a homozygous transition c.496C>T in exon 6.

From our patient group, there was a single affected individual whose whole blood was submitted to our laboratory for molecular genetic diagnostic testing. By direct sequencing, the patient was shown to be homozygous for a novel intronic splice-donor site mutation located in intron 13 of *ASL *(IVS13+5G>C). In addition, the parents of this patient were shown to be heterozygous carriers for this mutation, confirming segregation of the mutation in an autosomal recessive pattern of inheritance. However, to confirm that this splice-site mutation in intron 13 alters splicing, a cDNA fragment was amplified and directly sequenced in the index patient and an unrelated control sample that encompassed a part of exon 12 to part of exon 14. A band of 222 bp was detected in the normal control sample whereas subsequent mRNA analysis of the patient revealed a band of 138 bp. The predicted normal size of exon 13 is 83 bp and this exact difference in size between the normal control and patient fragments is highly indicative that exon 13 is completely spliced-out in the patient and hence confirms that this mutation does affect normal splicing.

One patient was homozygous for a c. 544 C>T transition, which resulted in the previously reported nonsense mutation R182X [11], in exon 7 of *ASL*, predicting premature truncation of the normal 460 amino acid ASL product at position 182.

Finally, one patient was homozygous for a transversion, c.1081G>T, also resulting in a novel nonsense mutation, G361X which also predicts premature truncation of the normal amino acid ASL product. The one novel nonsense and four missense mutations were confirmed by their absence in ~300 chromosomes from the normal population. The presence of these mutations was confirmed in parental DNA when available.

**Figure 1:**
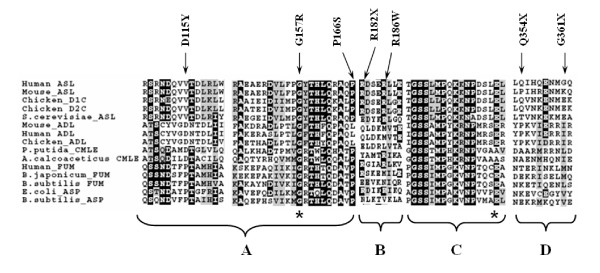
**Consensus sequences of the ASL gene superfamily**. The alignment shading corresponds to consensus of 100% (black) and 80% (dark gray). (A) corresponds to amino acid conserved domain I containing the novel mutations D115Y, G157R and P166S. (B) corresponds to amino acid conserved domain II containing the nonsense mutations R182X, and R186W. (C) corresponds to amino acid conserved domain III. (D) corresponds to the carboxy terminal containing Q354X and the novel G361X mutations. The '↓' represents the position of the mutation. The '*' represents the putative catalytic residues. FUM, fumarase; ASP, ammonia-aspartate lyase; ADL, adenylosuccinate lyase; CMLE, 3-carboxy-*cis, cis*-muconate lactonizing enzyme; D2C, δ2 crystallin; D1C, δ2 crystallin; ASL, argininosuccinate lyase. The alignment was performed using the ClustalW program (GenomeNet).

To further investigate the five novel *ASL *coding variants, multiple sequence alignment (protein) was performed using the ClustalW program (GenomeNet) to determine cross-species conservation of *ASL *at the position of each novel mutation (Figure 1). Strong cross-species conservation of *ASL *was observed at the sites in all 4 of the novel missense mutations. However, amino acid variation was noted at residues (R182X, Q354X and G361X) where nonsense mutation resulted in premature truncation.

## Discussion

Although approximately 50 mutations underlying ASA have been reported to date, apparently only three have been identified in Arab patients, one being the result of a previous study by our group[8,15]. In this study with the exception of one patient, a compound heterozygote for the R186W and D115Y mutations, all patients had homoallelic mutations reflecting consanguinity in the Saudi population. Four of the novel mutations identified by this study occur in highly conserved domains of ASL and indeed other members of the ASL superfamily such as class II fumarase [16], adenylosuccinate lyase [17], L-aspartase [16,18], 3-carboxy-cis, cis-muconate lactonizing enzyme (CMLE) [19], and δ crystalline [20,21] (Figure 1). Whilst overall amino acid sequence similarity of these enzymes is low (<20%), three regions of highly conserved residues across the ASL superfamily represent consensus sequences which are involved in the catalytic mechanism of these enzymes [22]. The crystal structure of ASL like five members of the ASL superfamily includes a common protein fold where monomers containing three structural domains form dimers which associate to form a tetramer [23]. The superfamily consensus sequences are spatially removed from each other in the monomers, but come together at each of the four corners of the tetramer to form four active site clefts. Mutations within the superfamily consensus sequences are highly likely to alter active site clefts to the detriment of enzymatic activity and offer explanation for the highly conserved nature of these regions. The R186W mutation which results in the substitution of arginine by tryptophan occurs 3 residues upstream of the putative catalytic residue of the central ASL superfamily consensus domain and predicts pathogenicity of this change consistent with its rare occurrence in the general population. A second novel mutation D115Y, identified by this study occurs in the amino ASL superfamily consensus domain once again consistent with pathogenicity related to alteration of the active cleft. A third missense mutation G157R occurs between the amino and central consensus domains with no immediately apparent functional significance. However, this alteration was not observed in ~300 chromosomes from the ethnically matched normal population. Three nonsense mutations were identified among Saudi ASAuria patients, two from this study (R182X and G361X), the other having being previously reported [15].

The homotetrameric structure of ASL introduces the possibility of clinical heterogeneity resulting from interallelic complementation. However, given extensive consanguinity in the Saudi population and as evidenced by this study, homoallelic mutations are almost universally observed in this group negating clinical heterogeneity resulting from interallelic complementation. Only one compound heterozygote was identified in this study and had mutations in the amino and central ASL superfamily consensus domains. The clinical consequence of this and the role of complementation could not be assessed since all patient samples were fully anonymized as part of IRB approval.

Together the eight mutations described in this and our previous study, cover above 90% of the underlying mutations of *ASL *in ASAuria patients of Saudi ethnicity. The two most common (Q354X and R186W) cover 89% of the population with others likely to represent private familial mutations. This coverage provides an efficient molecular diagnosis of ASAuria in the Saudi population and lays the foundation for preventative measures including inductive screening of extended families, counseling, and regional pre-marital screening. In addition, identification of these mutations enables provision of pre-implantation and prenatal diagnosis as appropriate. As a result of this and a previous study [15], substantial reduction in live births with ASAuria in the Saudi population is possible. The common mutation identified by this study may also be more widely relevant in the Arab world.

## Competing interests

The authors declare that they have no competing interests.

## Authors' contributions

FI was primarily responsible for the design, molecular genetic studies, data interpretation, drafting and finalizing the manuscript; MAS was primary responsible for clinical support and sample collection; DT and BAM assisted in carrying out molecular genetic studies; OA participated in methodology; MSR contributed with sample collection and biochemical support; BFM participated in design, data interpretation and final editing of the manuscript. All authors have read and approved the final manuscript.
